# Topical Opioids for Pain in Malignant and Non-Malignant Chronic Ulcers: A Systematic Review

**DOI:** 10.51894/001c.164357

**Published:** 2026-07-31

**Authors:** Cooper Myers, Jennifer Zora, Dema Boutany, Anthony Eid, Ferdous Nipu, Bassil Darwich, Jeremy Kramer

**Affiliations:** 1 Henry Ford Warren Hospital

## 32

### Background

For over thirty years, clinicians in Europe and Australia have turned to topical opioids like morphine and diamorphine to manage the severe pain of malignant fungating wounds and refractory chronic ulcers. Yet, despite this clinical longevity, the evidence remains surprisingly thin and inconsistent. While certain reports highlight rapid analgesia without the burden of systemic side effects, other data fail to show any real benefit over placebo in non-malignant wound populations.

### Objectives

This systematic review evaluates the analgesic efficacy, duration of effect, opioid-sparing potential, and safety of topical morphine, diamorphine, and methadone for painful chronic ulcers, focusing on both malignant and non-malignant etiologies.

### Methods

The systematic search included MEDLINE, Embase, CINAHL, Cochrane CENTRAL, and grey literature without language restrictions, adhering to PRISMA guidelines. The review included RCTs, non-randomized trials, cohorts, and case series (n ≥ 5) reporting pain outcomes. The primary outcomes were change in pain intensity and the proportion of patients achieving a reduction in pain. We evaluated risk of bias using RoB-2 and ROBINS-I and assessed certainty of evidence through GRADE.

### Results

The search yielded 10 eligible studies (7 RCTs, 1 open-label pilot, 1 cohort, and 1 case series) totaling 165 patients. Efficacy data were stratified by wound type. Moderate-certainty evidence supports meaningful pain reduction in malignant wounds; for instance, NRS scores dropped from 5.9 to 2.5 over 28 days in one cohort. Conversely, evidence for non-malignant ulcers was categorized as very low certainty, showing only sporadic, short-lived benefits. Three studies hinted at an opioid-sparing effect, though this remained low-certainty. Safety profiles were excellent across the board; adverse events were strictly localized, and systemic absorption was negligible.

### Conclusion

The evidence for topical opioids in wound care is both limited and highly dependent on the underlying etiology. While they appear to be a safe, moderate-certainty option for malignant or palliative lesions, their routine use in non-malignant ulcers isn’t supported by the current data. There is a need of rigorous, larger-scale RCTs with standardized endpoints to move this practice beyond individualized, off-label use and settle the ongoing clinical debate.

